# Symptom Persistence Following COVID-19 Infection among an Indigenous Community Residing in the Isthmus of Tehuantepec, Oaxaca, Mexico

**DOI:** 10.3390/jcm13175310

**Published:** 2024-09-07

**Authors:** Araceli Guerra-Martínez, Iván Antonio García-Montalvo, Aurelia Guerra-Martínez, Héctor Martínez Ruíz, Diana Matías-Pérez, Eduardo Pérez-Campos, Roberto Ariel Abeldaño Zuñiga

**Affiliations:** 1 PostGraduate Studies and Research, National Technological Institute of México/ITO, Oaxaca (TecNM), Oaxaca 68030, Mexico; aguerraguerra999@gmail.com (A.G.-M.); ivan.garcia@itoaxaca.edu.mx (I.A.G.-M.); dian_1007@hotmail.com (D.M.-P.); perezcampos123@yahoo.es (E.P.-C.); 2 Ministry of Health of the State of Oaxaca, Morelos 62508, Mexico; aureguerra@gmail.com; 3 Research Center Faculty of Medicine National Autonomous University of Mexico—Autonomous University "Benito Juárez" of Oaxaca, Faculty of Medicine and Surgery, Oaxaca 68120, Mexico; drheccctor@hotmail.com; 4 Faculty of Medicine and Surgery, Autonomous University "Benito Juárez" of Oaxaca, Oaxaca 68120, Mexico; 5 General Hospital “Aurelio Valdivieso MD” SS, Oaxaca 68040, Mexico; 6 Mexican Social Security Institute, General Zone Hospital No. 1 “Demetrio Mayoral Pardo”, Oaxaca 68000, Mexico; 7 Clinical Pathology Laboratory, “Eduardo Pérez Ortega”, Oaxaca 68000, Mexico; 8 Yhteiskuntadatatieteen Keskus, Valtiotieteeellinen Tiedekunta, Helsingin Yliopisto, 00100 Helsinki, Finland; 9 PostGraduate Department, University of Sierra Sur, Oaxaca 70800, Mexico

**Keywords:** post-COVID-19, pandemic, COVID-19, Oaxaca, Isthmus of Tehuantepec

## Abstract

**Introduction/Objectives:** Several studies have documented the development and persistence of symptoms related to COVID-19 and its secondary complications up to 12 months after the infection. We aimed to identify the medical complications following COVID-19 infection in the Indigenous Zapotec population of the Isthmus of Tehuantepec region in Oaxaca, Mexico. **Methods:** This is a cross-sectional analytical study that included 90 Indigenous Zapotec participants (30 males and 60 females) from the Tehuantepec region, Oaxaca, Mexico, who had an infectious process due to SARS-CoV-2. Sociodemographic and clinical data were identified through questionnaires. **Results:** Among the 201 participants, 90 individuals (66.7% women, 33.3% men) had contracted COVID-19. Out of these, 61 individuals reported persistent symptoms post-infection, with a mean symptom duration of 13.87 months. The results show significant variations in symptom duration based on age, marital status, educational attainment, vaccination status, and blood group. The most commonly reported symptoms included a dry cough, fever, myalgia, fatigue, headache, and depressive symptoms. **Conclusions:** This study highlights the post-COVID-19 symptoms and their prevalence within a specific sample of the Indigenous Zapotec population in Oaxaca, along with the sociodemographic and clinical factors influencing the duration of these symptoms. It underscores the necessity of personalized recovery strategies and highlights the critical role of vaccination in mitigating the long-term impacts of SARS-CoV-2.

## 1. Introduction

The outbreak of coronavirus disease in 2019 (COVID-19), which began in Wuhan in December 2019 [1], was declared an international public health emergency in January 2020 and a pandemic in March 2020 due to its rapid global spread [2]. Some studies have documented the adverse effects of the COVID-19 pandemic worldwide; however, the findings are still inconclusive [3,4,5,6].

In Mexico, household incomes decreased by an average of approximately 48.5% during the first months of the pandemic. Households spent an average of USD 400 per month on healthcare; in addition, wealthier households spent more on healthcare, with a greater proportion allocated to hospital care, while poorer households allocated a higher proportion to primary care and the purchase of medications [7].

In 2020, approximately 56% of the national population sought care from private health services. Among this percentage, the proportion from rural areas was slightly higher. The primary reasons for choosing private services were as follows: 29% reported having affiliation or coverage, 22.4% cited proximity to their homes, and 11.6% indicated low cost. However, the percentage of the rural population choosing services based on affiliation was 18.8%, which is lower than that found in urban areas (32%). Proximity (25.8%) and low cost (15.4%) were more significant factors in rural locations [7].

Similarly, on average, the travel time from home to the nearest health facility is significantly longer in rural areas (40.2 min) compared to urban areas (27.2 min). Regarding the waiting time at the facility, rural populations experience longer waits (42.1 min) compared to urban populations (38.1 min). Among the regions of the country, the South Pacific region reported the longest average waiting time, at 50.1 min [7].

The Isthmus of Tehuantepec region in Oaxaca, covering 1,997,557 hectares, is known for its agricultural, fishing, and salt production activities. It is notable for its wind energy potential, strategic location, and development projects. However, it faces challenges related to housing, basic services, and seismic activity [8]. The inhabitants of this region have dietary habits characterized by a high consumption of carbonated beverages, alcohol, tobacco, coffee, and salt. Women in this region are more obese than men, and it has been observed that rural origin, night-time food consumption, and a family history of chronic degenerative diseases are statistically significant factors in this population [9]. Overweight and obesity in the youth of this region are significantly associated with the presence of risky eating behaviors. Similarly, young women show a higher prevalence of these conditions [10].

Coronaviruses are enveloped viruses with single-stranded, positive-sense ribonucleic acid (RNA) and are named for their crown-like appearance in electron micrographs. SARS-CoV-2 belongs to the betacoronavirus subgenus, similar to the virus responsible for severe acute respiratory syndrome (SARS). This virus proves the high epidemic potential characteristic of coronaviruses [11]. Following COVID-19 infection, studies have observed prolonged symptoms and secondary complications associated with the disease. This condition, termed ‘post-COVID-19 condition,’ encompasses a range of symptoms affecting all organ systems, with primary manifestations in the neuropsychiatric, respiratory, and cardiac domains [12].

According to a cohort study of 1733 patients in a Wuhan hospital, the most common symptoms after COVID-19 were muscle fatigue/weakness in 63% (2370 of 3762), sleep disturbance in 26% (978 of 3762), and anxiety/depression in 23% (865 of 3762). However, less than 10% (376 of 3762) reported issues with mobility, daily activities, or personal care. Multivariate regression analysis found associations between the severity of hospitalization and abnormal pulmonary diffusion tests, chest imaging abnormalities, and anxiety or depression. Women were found to be at increased risk for pulmonary abnormalities, anxiety, and fatigue or muscle weakness. Consequently, the study concluded that women may be a primary target population for long-term recovery interventions [13].

Davis et al. [14] conducted an international web-based survey with 3762 participants from 56 countries, of whom 8.4% reported hospitalization. The survey found 205 different symptoms and tracked 66 of these over a period of seven months. The most prevalent symptoms after six months were fatigue (77.7%), post-exertional malaise (72.2%), and cognitive dysfunction (55.4%). The study concluded that a significant proportion of patients do not recover within seven months and continue to experience a substantial burden of symptoms [14].

Additionally, in a prospective study conducted by Carfi et al. involving 143 Italian patients hospitalized with COVID-19, 83% continued to experience at least one symptom an average of 60 days after discharge. The most common persistent symptoms were fatigue (53.1%), dyspnea (43.4%), joint pain (27.3%), and chest pain (21.7%) [15]. Similarly, a study in Switzerland of 669 confirmed COVID-19 patients (primarily outpatients) found that 32% continued to experience at least one symptom, such as fatigue, dyspnea, or loss of taste, an average of 43 days after discharge [16].

Drawing from these and other studies, the prevalent persistent symptoms following acute COVID-19 include fatigue, dyspnea, chest discomfort, persistent cough, anxiety, depression, post-traumatic stress disorder, and cognitive impairments such as impaired memory and concentration (Table 1).

The results of 17 studies (see Table 1), encompassing a total of 294,443 participants across 57 countries, revealed the following: 130,378 were male and 163,990 were female, with a mean age of 49.6 years, ranging from 6 to 85 years. Those findings did not report data on COVID-19 recurrence and indicate that the predominant persistent symptoms up to 12 months after COVID-19 infection are fatigue (mean prevalence 37.6%), dyspnea (mean prevalence 25.4%), myalgia (mean prevalence 10.6%), cough (mean prevalence 11.49%), headache (mean prevalence 11.36%), chest pain (mean prevalence 12.49%), arthralgia (mean prevalence 14.11%), anosmia (mean prevalence 5.15%), dysgeusia (mean prevalence 6.86%), and anxiety or irritability (mean prevalence 19.85%). On average, 43.06% of participants reported no symptoms following COVID-19 infection.

These studies have linked the severity of infection, age [29], female sex, and the presence of comorbidities with the development of post-COVID-19 condition [18,19,29,30,31]. Additionally, factors such as the duration of hospital stay [26], admission to the ICU [29], hypertension, active smoking [25], and the number of symptoms experienced during the course of the illness have also been associated with this condition [23,24].

Some studies show that age, disease severity, and the time to viral resolution are not associated with the development of post-COVID-19 condition [25,27]. On the other hand, women are at a higher risk of developing anxiety, depression, or poor sleep quality following COVID-19 compared to men [17,20,21]. Pre-existing chronic pulmonary disease has been associated with chronic fatigue, while asthma is linked to a higher risk of developing neurological symptoms [30].

Fatigue, identified as the primary symptom following COVID-19 infection, has been reported as the most prevalent symptom. Female sex is significantly associated with experiencing three or more symptoms, including fatigue [17,25].

Patients whose infection severity led to hospitalization or admission to the Intensive Care Unit (ICU), as well as elderly patients, show a significantly higher incidence of cognitive difficulties. However, those who were hospitalized are less likely to experience myalgia or headaches [19,20].

Regarding headaches, it was found that female sex is not significantly associated with the onset of this symptom at hospital admission [17]. However, women and younger individuals are significantly more likely to experience headaches following infection [19]. Patients who experienced fever, dyspnea, cough, and lethargy during COVID-19 infection are at a higher risk of developing post-COVID-19 condition compared to those with other associated symptoms [18]. Additionally, a decline in pulmonary function was observed in recovered patients, suggesting a potential for developing pulmonary fibrosis [26,27].

On the other hand, a sore throat was not associated with the development of post-COVID-19 condition [20]. Similarly, no association was observed between age and the prevalence of fatigue, muscle weakness, anxiety, or depression [22].

In general, the prevalence of 203 symptoms across 10 body systems was estimated. Systemic and neurological/cognitive symptoms are the most prevalent [14], with some of these symptoms not being present before, during, or at the time of discharge [30]. Regarding medical complications, a group of subjects were diagnosed with diabetes following their COVID-19 illness [18,22]. The duration of symptoms associated with post-COVID-19 condition has been examined in studies by Huang et al. and Kikkenborg Berg et al., which report a duration of up to 12 months [22,28]. Overall, studies show an average duration of 5.1 months.

The COVID-19 pandemic [32], as noted by the World Health Organization (WHO), has significantly affected global gains in life expectancy. From 2019 to 2021, life expectancy decreased by 1.8 years to 71.4 years, with healthy life expectancy also falling by 1.5 years to 61.9 years in 2021. These impacts have varied globally, as follows: in the Americas and Southeast Asia regions, life expectancy dropped by 3 years, and healthy life expectancy by 2.5 years; whereas, in the Western Pacific region, the declines were minimal, with less than 0.1 years lost in life expectancy and 0.2 years in healthy life expectancy [33]. This study aimed to identify the primary medical complications following COVID-19 infection in a sample of Indigenous Zapotec participants from the Isthmus of Tehuantepec region in Oaxaca, Mexico.

## 2. Methods

### 2.1. Description of the Population

The state of Oaxaca is one of the 32 states in the Mexican Republic, renowned for its rich cultural diversity, which is reflected in its numerous ethnic groups and the 1.22 million speakers of indigenous languages. The predominant ethnic group is Zapotec. Located in the southwest of Mexico, Oaxaca has a population of 4,132,148 inhabitants, with 47.8% men and 52.2% women [34].

Oaxaca is divided into eight geographic and cultural regions. Among these, the Isthmus of Tehuantepec—the region analyzed in this study—holds strategic importance as the narrowest point in the country, easing communication between the Pacific Ocean and the Gulf of Mexico [35]. This region is home to a multi-ethnic population, predominantly Zapotec, with various ethnic groups each contributing distinct histories and languages [36,37]. The climate in this region is generally dry and warm, with moderately fertile soils and an average altitude of 650 meters above sea level [35].

Historically, the Isthmus of Tehuantepec has been a focal point of disasters such as floods [10], earthquakes [38], epidemics [39], and, most recently, the COVID-19 pandemic [40]. In addition to its social, political, and cultural dimensions that affect the towns in this region, factors such as nutrition significantly affect the quality of life of the population [10].

### 2.2. Study Design

A cross-sectional and analytical study was conducted in populations from the Isthmus of Tehuantepec, Oaxaca.

### 2.3. Participants

This study was conducted with the collaboration of three physicians, two nurses, three assistants, and one researcher. The physicians previously trained the assistants to take anthropometric measurements and correctly use the following instruments: measuring tape, TANITA scale, and stadiometer.

The sampling was purposive, targeting a population with a history of Indigenous identity. For participant recruitment, detection campaigns were organized at various locations in the Isthmus starting at 7:00 hours to take fasting plasma glucose (FPG) measurements. Community loudspeakers were used to call people to attend, and passers-by were also invited. The selection criteria were applied by the medical staff during interviews with the participants. The inclusion criteria were individuals over 18 years of age with no history of ischemic stroke (as such patients may present with marked neurological impairment [41]), and whose PCR, rapid antigen test, or rapid blood test results were positive for SARS-CoV-2.

**Figure 1:**
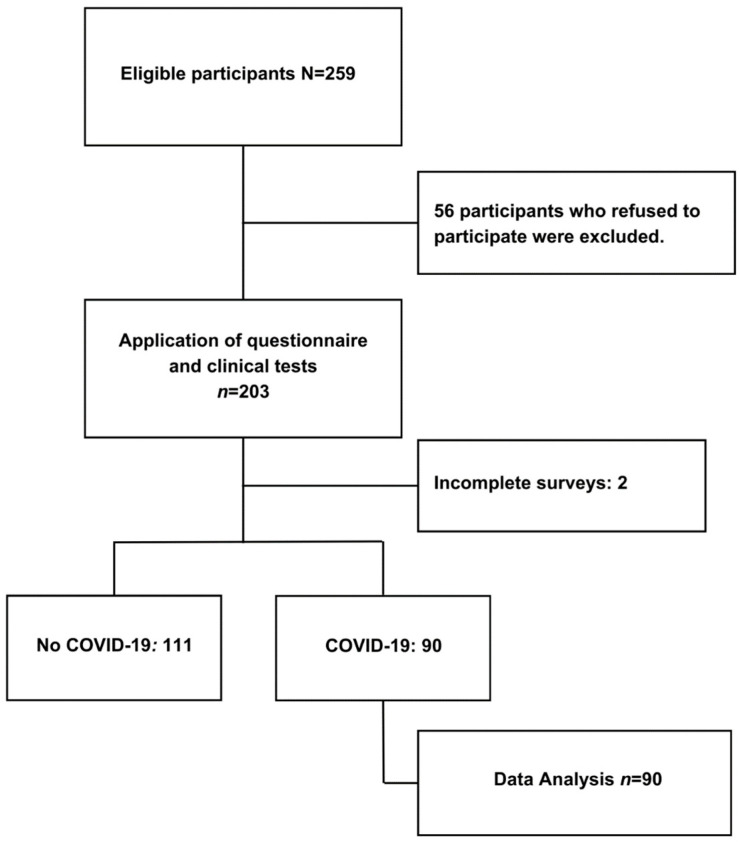
Participant selection flowchart.

The exclusion criteria were applied to participants who did not complete their questionnaire or decided to withdraw from the study at any point during the process. The final sample consisted of 90 participant records (Figure 1).

The participants signed an informed consent form before starting the study, and their data were protected, with records identified by consecutive numbers. These numbers were maintained and recorded during data processing as the sole means of patient identification. Additionally, only the researcher had access to the physical records of the participants, which will be securely destroyed once they are no longer needed for this study, thus preventing any risk of accidental disclosure or unauthorized access.

During the fieldwork, interviews were conducted in person, and questionnaires on sociodemographic data and clinical history were administered. Additionally, fasting plasma glucose (FPG) [42] and blood glucose (BPG) tests were conducted following the criteria and recommendations of the American Diabetes Association (ADA), and blood pressure was measured based on the ACC/AHA American College of Cardiology Guidelines [43]. Other measures included grip strength in the non-dominant hand [44], oxygen saturation [45], respiratory rate [46], heart rate [47], temperature [48], and body mass index using Tanita scales [49]. Seventy-seven percent of the participants were speakers of an Indigenous language, needing the assistance of interpreters and ensuring that collaborating medical personnel were also speakers of an indigenous language.

### 2.4. Dependent Variables

The dependent variables of the present study are the condition after COVID-19, which refers to the presence or absence of symptoms following COVID-19 infection (1 = Yes, 0 = No), and the duration of the symptoms after COVID-19, which refers to the time (in days and months) determined by the following formula:

Duration of symptoms=SD−DOS

where:

SD: Survey date.
DOS: Date of onset of symptoms associated with COVID-19.

To evaluate the association of the variable ‘Duration of symptoms after COVID-19’ with other variables, the categories short-term (1–6 months), medium-term (7–12 months), and long-term (>12 months) were generated.

### 2.5. Explanatory Variables

The explanatory variables were as follows: sex, health coverage, age, age group, marital status, education level, identity, native language speaker, head of household, occupation, ethnic group, indigenous language, blood group, COVID-19 vaccination status, vaccination before COVID-19, recurrent COVID-19 infection, severity of infection, type of COVID-19 vaccine, influenza vaccine after COVID-19, and influenza vaccine before COVID-19. Regarding the influenza vaccine, it was recorded whether participants had received it before or after their COVID-19 infection.

The definition used for the variable ‘severity of infection’ was proposed by Rodríguez-Morales, et al., who define the severity of infection as follows: mild (without evidence of viral pneumonia or hypoxia), moderate (with clinical signs of pneumonia but without signs of severe pneumonia), severe (with clinical signs of pneumonia, severe respiratory distress, or SpO2 < 90%), and critical (with clinical signs of acute respiratory distress syndrome (ARDS)) [50].

### 2.6. Data Analysis

In this study, data were analyzed using SPSS version 29.0.2.0 and JASP version 0.17.1 software, employing descriptive and inferential methods to explore and assess the data. Records with more than 10% missing values were excluded for being incomplete and, therefore, ineligible for inclusion in the study sample. Categorical variables were coded as factors, and continuous variables were standardized. For descriptive analysis, basic statistics were calculated for all sociodemographic and clinical variables. Frequencies and percentages for categorical variables are detailed in Table 2 and Table 3. The Chi-square test was used to evaluate the associations between explanatory variables and the dependent variable.

### 2.7. Ethical Considerations

Before starting the study, approval was obtained from the Ethics Committee of the Technological Institute of Oaxaca. The research protocol was submitted for review and approval by the committee to ensure that it was following the ethical principles necessary for its development. The IRB approval code is: 12/09/2023-001.

The participants were provided with printed consent forms and promptly informed about the research’s aims, the procedures involved, the potential risks and benefits, and their right to withdraw from the study at any time without facing any penalties. Only those who signed this consent form entered the study group. Confidentiality of the information provided by the participants was guaranteed, and their data were anonymized and coded with a sequential number to prevent personal identification.

Additionally, all necessary measures were taken to minimize any potential risk to the health or integrity of the participants. All potential physical, psychological, and social risks associated with participation were assessed and mitigated.

## 3. Results

The sample comprised 90 participants, all with confirmed cases of COVID-19. Among these, 66.7% were women and 33.3% were men. The mean age of the subjects was 47.75 years, with a range from 19 to 85 years. The predominant age group was 40 to 59 years. Additionally, 61 participants experienced prolonged symptoms after COVID-19 infection. In this group, the mean age was 51.15 years (see Table 4). Of these, 67.21% were married, 32.78% had attended primary school, and 96.72% were identified as belonging to an ethnic group, with the Zapotec ethnic group being the most prominent, at 50.81%. Furthermore, 60.65% spoke a native language. Among these participants, 54.09% were engaged in housework and 16.39% were employed (see Table 2). The mean duration of symptoms was observed to be 13.87 months, with a maximum of 35 months (Table 4).

A statistically significant difference was observed in the duration of symptoms based on the participants’ age (*p* = 0.002). Among individuals aged 15 to 30 years, 77.78% experienced symptoms of a very long-term duration. In the 31 to 45 years age group, 73.33% reported very long-term symptoms, while 55.56% of those aged 46 to 60 years experienced very long-term symptoms. For participants over 60 years, 73.68% showed medium-term symptoms, and 21.05% had very long-term symptoms (Table 4).

Similarly, marital status was significantly associated with the duration of symptoms (*p* = 0.01). Among single individuals, 78.57% experienced very long-term symptoms. In contrast, married individuals included 48.78% with medium-term symptoms, 7.32% with long-term symptoms, and 43.90% with very long-term symptoms. Divorced and cohabiting individuals also predominantly experienced very long-term symptoms, albeit with smaller variations compared to the other groups (Table 5).

Schooling was statistically significantly associated with the duration of symptoms (*p* < 0.001). In individuals with elementary education, 65% reported experiencing medium-term symptoms and 35% very long-term symptoms. The participants with secondary education, high school, and bachelor’s degrees showed a tendency towards very long-term symptoms, with percentages of 76.92%, 66.67%, and 77.78%, respectively. Additionally, the patients with no formal education had a higher prevalence of medium-term symptoms, reported at 85.71% (Table 5).

Influenza vaccination prior to COVID-19 was significantly associated with the duration of symptoms (*p* = 0.007). Of the participants who were not vaccinated, 54.84% reported experiencing medium-term symptoms, while 66.67% of those who were vaccinated experienced very long-term symptoms. Similarly, COVID-19 vaccination prior to infection also showed significant differences (*p* = 0.02). Among non-vaccinated participants, 78.95% reported very long-term symptoms, while 45.24% of vaccinated individuals experienced medium-term symptoms and 40.48% experienced very long-term symptoms (Table 5).

Regarding the differences observed among blood groups, statistical significance was noted (*p* = 0.006). Individuals with blood group O+ showed a higher prevalence of very long-term symptoms (73.91%), while those with other blood groups, who did not respond, predominantly experienced medium-term symptoms (54.29%) (Table 5).

The analysis of the duration of COVID-19 symptoms revealed significant differences (*p* = 0.07) between the long-term and short-term groups for certain clinical variables. Specifically, fatigue was more prevalent in the long-term group. Muscle pain also occurred significantly more frequently in the long-term group (*p* = 0.006). Conversely, dry cough was more common in the short-term group (*p* = 0.001). In addition, headache was more prevalent in the long-term group, with a *p*-value of 0.004 (Table 6).

Similarly, statistical differences were observed for fever, which was more prevalent in the short-term group (*p* = 0.001). In contrast, depression was significantly more common in the long-term group (*p* = 0.002). Additionally, skin rashes were significantly more frequent in the long-term group (*p* = 0.02) (Table 6).

## 4. Discussion

This study aimed to identify the primary medical complications following COVID-19 infection within the Indigenous Zapotec population of the Isthmus of Tehuantepec region in Oaxaca, Mexico. It provides insight into the symptoms that persist after COVID-19 infection, commonly referred to as post-COVID-19 condition. This condition has garnered considerable attention from the scientific community due to its impact on patients’ quality of life and its potential implications for the quality of medical care within this specific population subgroup. Studying this condition in communities historically affected by poverty and inequality, such as those in the Isthmus of Tehuantepec, is particularly critical, as these challenges have been exacerbated by the pandemic.

The results from this study align with previous research regarding the persistence of symptoms following SARS-CoV-2 infection. The findings show a complex interaction among various physiological systems, including the respiratory, cardiovascular, immune, and central nervous systems. However, this study reveals a divergence from earlier studies [14,15,17,18,19,20,21,22,23,24,25,26,27,28,29,30,31], which found fatigue as the most frequently reported symptom. In contrast, this research found that a dry cough was the most prevalent symptom among participants, followed by fatigue.

Additionally, while previous studies reported a maximum symptom duration of 12 months, this study found a mean duration of 13.87 months and a maximum of 35 months. This discrepancy highlights the need for implementing health strategies that extend beyond the acute phase of COVID-19 to address the post-recovery period, especially in Indigenous communities like the Zapotec, where the prevalence of symptoms of the disease appears more prolonged and complex. Compared with other international studies, these findings align with the prevalence of prolonged symptoms such as fatigue and dyspnea. However, discrepancies were noted in the prevalence of other symptoms, such as chest pain and cognitive dysfunction.

These variations may be attributed to differences in the target populations and the methods employed. It is particularly important to consider the cultural characteristics of the Indigenous Zapotec population from the Isthmus of Tehuantepec, Oaxaca, México. This population is globally renowned for its rich traditions, including the active participation of women in social and political spheres. Historically, women in this region have played a crucial role in education, health, the economy, and, in general, all aspects of family life [51], which could influence the prevalence and reporting of certain post-COVID-19 symptoms.

The findings by Huang suggest that women may be the primary target for long-term recovery interventions [22], and this study also found a higher prevalence of symptoms among women post-COVID-19. This could be attributed to the historical role of women in overseeing healthcare and providing attention within this region, which could elucidate the findings observed in this study and others [52,53]. This study made a deliberate effort to include a larger number of male participants, particularly from police and fire departments, to ensure a more comprehensive representation of how COVID-19 affects different genders within this specific community.

Moreover, the dietary patterns of the Indigenous Zapotec population in the Isthmus of Tehuantepec, marked by substantial intake of carbonated beverages, alcohol, tobacco, coffee, and salt, combined with elevated obesity rates, particularly among women [9], may significantly influence the health outcomes associated with this condition. This study documented a mean body mass index (BMI) of 28.81, with a range spanning from 16.4 to 52.6, indicating a high prevalence of overweight and obesity within this study. These elevated obesity rates, in conjunction with deleterious dietary behaviors, are likely to exacerbate both the severity and duration persistent symptoms. Obesity is known to impair immune function and heighten the susceptibility to chronic diseases, which can impede recovery from viral infections and result in more severe or prolonged symptoms.

The findings of this study suggest that the presence of comorbidities appears to be a significant factor in the development of this condition. These findings are consistent with the existing literature, which highlights that individuals with pre-existing health conditions are at a higher risk for prolonged symptoms following infection [18,19,29,30,31]. In the context of the Indigenous Zapotec population from the Isthmus of Tehuantepec, these results are particularly pertinent, given the unique dietary patterns and high prevalence of obesity within this group. These results underscore the necessity for targeted healthcare interventions and comprehensive monitoring for individuals with comorbidities and high obesity rates, particularly in Indigenous populations where cultural and lifestyle factors significantly impact health outcomes, such as among the Zapotecs.

It is essential to acknowledge the limitations of this study, including the possibility that the sample may not fully represent all manifestations of COVID-19. Factors such as the timing of the study, which occurred during an ongoing pandemic, the method, the geographic location, and the specific characteristics of the populations studied all contribute to these limitations. A larger and more diverse sample would offer a more comprehensive understanding of the range of symptoms associated with this disease across different contexts, particularly in Indigenous populations where social and cultural dynamics can significantly influence health outcomes.

Finally, the global impact of the pandemic on life expectancy and healthy life expectancy highlights the enduring consequences of SARS-CoV-2 at both individual and population levels. There is a limited understanding of the effects of COVID-19 within Indigenous communities, underscoring the need for a comprehensive approach to fully grasp the disease’s impact across all levels.

## 5. Conclusions

This study highlights the prevalence and persistence of post-COVID-19 symptoms among a specific sample of Indigenous Zapotec participants from the state of Oaxaca. It also explores sociodemographic and clinical factors—including schooling, age, marital status, blood group, and vaccination status—that influence the duration of these symptoms. The findings underscore the need for comprehensive and personalized recovery strategies and emphasize the critical role of vaccination programs in mitigating the prevalence of symptoms of SARS-CoV-2.

## Figures and Tables

**Table 1:** Summary of main symptoms reported by participants that persist after hospital discharge in COVID-19 patients across various studies.

Symptoms	Frequency of Reports (%)																
	[17] %	[18] %	[19] %	[20] %	[21] %	[14] %	[22] %	[23] %	[24] %	[25] %	[26] %	[27] %	[28] %	[29] %	[30] %	[15] %	[31] %
Fatigue	61.30	72.8	12.82	33.00	63.00	77.74	20.00	38.30	11.27	39.50	69.00		0.57		2.00	53.10	44.81
Dyspnea	53.50	28.20		7.00		59.22		39.20	8.39	6.40	53.00	5.50	1.58	17.24	1.34	43.40	31.69
Myalgia			3.24	0.60	3.00	43.69	4.00	5.10	6.71	21.20		5.90		12.07	0.40		21.31
Dry cough				8.50		20.19		6.00	1.44		34.00	2.50	0.93	15.52	0.64		25.14
Headache		28.90	8.67	3.40	2.00	49.59	5.00	2.20					0.61		0.69		12.57
Chest pain **		28.90	12.60	0.80	5.00	56.21	7.00	4.40				0.40	0.15		0.34	21.70	
Arthralgia		31.40		1.40	9.00	37.06	12.00					5.90	0.23		0.96	27.30	15.85
Anosmia	4.05			2.00	11.00		4.00	6.30		4.80		4.60			0.34		9.29
Dysgeusia	2.70				7.00	25.16	3.00	4.70	3.84	5.30					0.19		9.84
Anxiety/irritability	15.6	38.00	22.82		23.00		26.00	9.20		18.80					1.10		
Rash	12.00			0.60	3.00	12.32	4.00						0.08		0.20		
Diarrhea	2.50				5.00	20.50						1.30		1.72	0.40		3.82
Odynophagia	2.50				4.00	20.60	3.00						0.11		0.15		
Hair loss	23.90				22.00		11.00	6.30							1.00		
Fever		11.10			<1	2.90							0.15	25.86			1.09
Cognitive dysfunction	9.60		7.88			55.49				20.20					1.30		
Lack of appetite					8.00	13.66	3.00						1.56		0.30		
Dizziness					6.00	27.02	5.00						1.69		0.54		
Depression	18.9	28.60								10.60	14.60	18.00			0.70		
Palpitation					9.00	40.89	9.00						1.18		0.70		

Source: Own elaboration. ** This category includes chest pain and chest tightness.

**Table 2:** Sociodemographic characteristics of participants (*n* = 90).

Variables	Frequency (*n* = 90)		Post-COVID-19 Condition			
			Yes		No	
	*n*	%	*n*	%	*n*	%
Sex						
Woman	60	66.7	43	47.8	17	18.9
Man	30	33.3	18	20.0	12	13.3
Marital Status						
Married	58	64.4	41	45.6	17	18.9
Single	21	23.3	14	15.6	7	7.8
Divorced	3	3.3	2	2.2	1	1.1
Separated	1	1.1	0	0.0	1	1.1
Cohabiting	3	3.3	1	1.1	2	2.2
Widower	4	4.4	3	3.3	1	1.1
Education						
Postgraduate	3	3.3	3	3.3	0	0.0
Undergraduate	12	13.3	6	6.7	6	6.7
High School	25	27.8	12	13.3	13	14.4
Secondary	18	20.0	13	14.4	5	5.6
Elementary	24	26.7	20	22.2	4	4.4
No studies	8	8.9	7	7.8	1	1.1
Identity						
Native	88	97.8	59	65.6	29	32.2
Native language speaker	55	61.1	37	41.1	18	20.0
Ethnic Group						
Zapotec	57	63.3	31	34.4	26	28.9
Chontal	24	26.7	22	24.4	2	2.2
Huave	5	5.6	5	5.6	0	0.0
Chinantec	1	1.1	1	1.1	0	0.0
Mixtec	0	0.0	0	0.0	0	0.0
None	3	3.3	2	2.2	1	1.1
Native language						
Zapotec	40	44.4	23	25.6	17	18.9
Chontal	10	11.1	10	11.1	0	0.0
Chinantec	1	1.1	1	1.1	0	0.0
Ümbeyajts	4	4.4	4	4.4	0	0.0
Mixtec	0	0.0	0	0.0	0	0.0
None	35	38.9	23	25.6	12	13.3
Occupation						
Domestic tasks	43	47.8	33	36.7	10	11.1
Employee	19	21.1	10	11.1	9	10.0
Merchant	6	6.7	4	4.4	2	2.2
Farmer	3	3.3	3	3.3	0	0.0
Firefighter	9	10.0	4	4.4	5	5.6
Student	4	4.4	3	3.3	1	1.1
Worker	0	0.0	0	0.0	0	0.0
Self-employed	3	3.3	3	3.3	0	0.0
Healthcare Worker	2	2.2	0	0.0	2	2.2
Unemployed	1	1.1	1	1.1	0	0.0
Retired	0	0.0	0	0.0	0	0.0
Age groups						
15–30	18	20.0	9	10.0	9	10.0
31–45	25	27.8	15	16.7	10	11.1
46–60	25	27.8	18	20.0	7	7.8
Over 60	22	24.4	19	21.1	3	3.3

Source: Results of surveys applied to study group.

**Table 3:** Clinical aspects and evolution of subjects who had COVID-19 (*n* = 90).

Variable	Frequency (*n* = 90)		Post-COVID-19 Condition				*p*
			Yes		No		
	*n*	%	*n*	%	*n*	%	
Comorbidities							
None	35	38.9	21	23.3	14	15.6	0.43
1	32	35.6	21	23.3	11	12.2	
2	15	16.7	13	14.4	2	2.2	
3	3	3.3	3	3.3	0	0.0	
4 or more	5	5.6	3	3.3	2	2.2	
Blood Type							
A+	11	12.2	2	2.2	9	10.0	<0.001
A negative	0	0.0	0	0.0	0	0.0	
AB+	0	0.0	0	0.0	0	0.0	
B+	1	1.1	1	1.1	0	0.0	
O+	40	44.4	23	25.6	17	18.9	
No response	38	42.2	35	38.9	3	3.3	
Influenza Vaccination							
Vaccinated	49	54.4	30	33.3	19	21.1	0.14
Not vaccinated	41	45.6	31	34.4	10	11.1	
COVID-19 vaccination							
Vaccinated	85	94.4	58	64.4	27	30.0	0.70
Not vaccinated	5	5.6	3	3.3	2	2.2	
Vaccine Type							
AstraZeneca	33	36.7	19	21.1	14	15.6	0.004
CanSino	41	45.6	35	38.9	6	6.7	
Pfizer	10	11.1	4	4.4	6	6.7	
Sputnik	1	1.1	0	0.0	1	1.1	
COVID-19 Severity *							
Mild: Symptomatic	66	73.3	43	47.8	23	25.6	0.04
Moderate: Pneumonia	15	16.7	14	15.6	1	1.1	
Severe: Severe pneumonia	8	8.9	3	3.3	5	5.6	
Critical: Septic shock	0	0.0	0	0.0	0	0.0	
Severe: ARDS **	1	1.1	1	1.1	0	0.0	
Management Type							
Outpatient	88	97.8	60	66.7	28	31.1	0.59
Hospitalized	2	2.2	1	1.1	1	1.1	
Intubated							
Yes	1	0.8	1	1.1	0	0.0	0.49
No	89	67.9	60	66.7	29	32.2	
ICU							
Yes	1	1.1	0	0.0	1	1.1	0.14
No	89	98.9	61	67.8	28	31.1	
Fasting Plasma Glucose							
Normal	33	36.7	22	24.4	11	12.2	0.99
Prediabetes	32	35.6	21	23.3	11	12.2	
Diabetes	25	27.8	18	20.0	7	7.8	

* To determine the severity of COVID-19 infection, the clinical case classification proposed by Rodríguez-Morales was used as a reference [50]. ** Acute respiratory distress syndrome. Source: Questionnaire application results.

**Table 4:** Descriptive statistics of clinical and demographic variables in participants with post-COVID-19 symptoms (*n* = 61).

Variables	Mean	Minimum	Maximum
Age (Years)	51.15	20	85
Oxygen saturation (SP_2_O) *	97.13	90	99
Mean blood pressure (mmHg)	89.85	70	127.33
Muscular strength (Kg)	53.54	20	130
Respiratory frequency (bpm)	19.67	16	30
Waist width (Centimeters)	96.52	74	128
Duration of symptoms post-COVID-19 (days)	418.97	25	1071
Duration of symptoms post-COVID-19 (months)	13.87	1	35
Body mass index (BMI)	28.81	16.4	52.6
Metabolic age (Years)	57.79	12	90
Number of comorbidities	1.16	0	6

Source: Results of surveys applied to study group. * Oxygen saturation was measured using a pulse oximeter, without supplementary oxygen.

**Table 5:** Association tests between the duration of symptoms and sociodemographic and clinical variables.

Variables		Duration of Symptoms						Total	*p*
		Short-Term (1–6 Months)		Medium-Term (7–12 Months)		Long-Term (>12 Months)			
		*n*	%	*n*	%	*n*	%		
Sex	Female	18	41.86	4	9.30	21	48.84	43	0.31
	Male	4	22.22	3	16.67	11	61.11	18	
	Total	22	36.07	7	11.48	32	52.46	61	
Health Coverage	IMSS	2	20.00	2	20.00	6	60.00	10	0.29
	ISSSTE	0	0.00	1	25.00	3	75.00	4	
	None	20	42.55	4	8.51	23	48.94	47	
	Total	22	36.07	7	11.48	32	52.46	61	
Age Groups	15–30	1	11.11	1	11.11	7	77.78	9	0.002
	31–45	1	6.67	3	20.00	11	73.33	15	
	46–60	6	33.33	2	11.11	10	55.56	18	
	Over 60	14	73.68	1	5.26	4	21.05	19	
	Total	22	36.07	7	11.48	32	52.46	61	
Marital Status	Single	1	7.14	2	14.29	11	78.57	14	0.01
	Married	20	48.78	3	7.32	18	43.90	41	
	Divorced	0	0.00	1	50.00	1	50.00	2	
	Cohabiting	0	0.00	1	100.00	0	0.00	1	
	Widowed	1	33.33	0	0.00	2	66.67	3	
	Total	22	36.07	7	11.48	32	52.46	61	
Education level	Primary	13	65.00	0	0.00	7	35.00	20	<0.001
	Secondary	1	7.69	2	15.38	10	76.92	13	
	High School	2	16.67	2	16.67	8	66.67	12	
	University Degree	0	0.00	2	22.22	7	77.78	9	
	No Education	6	85.71	1	14.29	0	0.00	7	
	Total	22	36.07	7	11.48	32	52.46	61	
Occupation	Domestic tasks	18	54.55	4	12.12	11	33.33	33	0.11
	Firefighter	1	25.00	0	0.00	3	75.00	4	
	Farmer	2	66.67	1	33.33	0	0.00	3	
	Merchant	0	0.00	0	0.00	4	100.00	4	
	Employee	1	11.11	1	11.11	7	77.78	9	
	Student	0	0.00	1	33.33	2	66.67	3	
	Factory Worker	0	0.00	0	0.00	1	100.00	1	
	Self-employed	0	0.00	0	0.00	3	100.00	3	
	Unemployed	0	0.00	0	0.00	1	100.00	1	
	Total	22	36.07	7	11.48	32	52.46	61	
Indigenous Origin	No	0	0.00	1	50.00	1	50.00	2	0.18
	Yes	22	37.29	6	10.17	31	52.54	59	
	Total	22	36.07	7	11.48	32	52.46	61	
Influenza Vaccination	No	17	54.84	2	6.45	12	38.71	31	0.007
(before COVID-19)	Yes	5	16.67	5	16.67	20	66.67	30	
	Total	22	36.07	7	11.48	32	52.46	61	
COVID-19 Vaccination	No	3	15.79	1	5.26	15	78.95	19	0.02
(prior to COVID-19)	Yes	19	45.24	6	14.29	17	40.48	42	
	Total	22	36.07	7	11.48	32	52.46	61	
Blood type	A+	0	0.00	1	50.00	1	50.00	2	0.006
	B+	1	100.00	0	0.00	0	0.00	1	
	O+	2	8.70	4	17.39	17	73.91	23	
	No response	19	54.29	2	5.71	14	40.00	35	
	Total	22	36.07	7	11.48	32	52.46	61	

Source: Questionnaire application results.

**Table 6:** Chi-square test results to evaluate the association between symptom duration and post-COVID-19 symptoms.

Variable		Symptom Duration (Groups)						Total (100%)	*p*
		Short-Term (1–6 Months)		Mid-Term (7–12 Months)		Long-Term (>12 Months)			
		*n*	%	*n*	%	*n*	%		
Chill	No	21	35.59	7	11.86	31	52.54	59	0.84
	Yes	1	50.00	0	0.00	1	50.00	2	
	Total	22	36.07	7	11.48	32	52.46	61	
Fatigue	No	20	44.44	5	11.11	20	44.44	45	0.07
	Yes	2	12.50	2	12.50	12	75.00	16	
	Total	22	36.07	7	11.48	32	52.46	61	
Shortness of breath	No	21	37.50	6	10.71	29	51.79	56	0.67
	Yes	1	20.00	1	20.00	3	60.00	5	
	Total	22	36.07	7	11.48	32	52.46	61	
Muscle pain	No	21	47.73	5	11.36	18	40.91	44	0.006
	Yes	1	5.88	2	11.76	14	82.35	17	
	Total	22	36.07	7	11.48	32	52.46	61	
Cough	No	3	8.57	6	17.14	26	74.29	35	<0.001
	Yes	19	73.08	1	3.85	6	23.08	26	
	Total	22	36.07	7	11.48	32	52.46	61	
Headache	No	21	46.67	6	13.33	18	40.00	45	0.004
	Yes	1	6.25	1	6.25	14	87.50	16	
	Total	22	36.07	7	11.48	32	52.46	61	
Chest pain	No	21	37.50	7	12.50	28	50.00	56	0.41
	Yes	1	20.00	0	0.00	4	80.00	5	
	Total	22	36.07	7	11.48	32	52.46	61	
Joint pain	No	21	38.18	6	10.91	28	50.91	55	0.58
	Yes	1	16.67	1	16.67	4	66.67	6	
	Total	22	36.07	7	11.48	32	52.46	61	
Loss of smell	No	21	35.00	7	11.67	32	53.33	60	0.41
	Yes	1	100.00	0	0.00	0	0.00	1	
	Total	22	36.07	7	11.48	32	52.46	61	
Altered taste	No	21	35.59	6	10.17	32	54.24	59	0.14
	Yes	1	50.00	1	50.00	0	0.00	2	
	Total	22	36.07	7	11.48	32	52.46	61	
Irritability	No	21	40.38	5	9.62	26	50.00	52	0.19
	Yes	1	11.11	2	22.22	6	66.67	9	
	Total	22	36.07	7	11.48	32	52.46	61	
Diarrhea	No	21	35.59	6	10.17	32	54.24	59	0.14
	Yes	1	50.00	1	50.00	0	0.00	2	
	Total	22	36.07	7	11.48	32	52.46	61	
Difficulty swallowing	No	21	35.59	7	11.86	31	52.54	59	0.84
	Yes	1	50.00	0	0.00	1	50.00	2	
	Total	22	36.07	7	11.48	32	52.46	61	
Hair loss	No	22	37.93	6	10.34	30	51.72	58	0.28
	Yes	0	0.00	1	33.33	2	66.67	3	
	Total	22	36.07	7	11.48	32	52.46	61	
Fever	No	3	7.69	6	15.38	30	76.92	39	<0.001
	Yes	19	86.36	1	4.55	2	9.09	22	
	Total	22	36.07	7	11.48	32	52.46	61	
Loss of appetite	No	22	36.67	7	11.67	31	51.67	60	0.63
	Yes	0	0.00	0	0.00	1	100.00	1	
	Total	22	36.07	7	11.48	32	52.46	61	
Nausea	No	22	37.93	7	12.07	29	50.00	58	0.24
	Yes	0	0.00	0	0.00	3	100.00	3	
	Total	22	36.07	7	11.48	32	52.46	61	
Depression	No	22	47.83	5	10.87	19	41.30	46	0.002
	Yes	0	0.00	2	13.33	13	86.67	15	
	Total	22	36.07	7	11.48	32	52.46	61	
Insomnia	No	22	39.29	6	10.71	28	50.00	56	0.21
	Yes	0	0.00	1	20.00	4	80.00	5	
	Total	22	36.07	7	11.48	32	52.46	61	
Sleep disturbances	No	22	37.93	7	12.07	29	50.00	58	0.24
	Yes	0	0.00	0	0.00	3	100.00	3	
	Total	22	36.07	7	11.48	32	52.46	61	
Blurred/double vision	No	22	37.29	6	10.17	31	52.54	59	0.180
	Yes	0	0.00	1	50.00	1	50.00	2	
	Total	22	36.07	7	11.48	32	52.46	61	
Vomiting	No	21	35.59	7	11.86	31	52.54	59	0.84
	Yes	1	50.00	0	0.00	1	50.00	2	
	Total	22	36.07	7	11.48	32	52.46	61	
Pain or discomfort	No	22	37.29	6	10.17	31	52.54	59	0.180
	Yes	0	0.00	1	50.00	1	50.00	2	
	Total	22	36.07	7	11.48	32	52.46	61	
Vertigo	No	22	36.67	7	11.67	31	51.67	60	0.63
	Yes	0	0.00	0	0.00	1	100.00	1	
	Total	22	36.07	7	11.48	32	52.46	61	
Abdominal symptoms	No	20	35.09	7	12.28	30	52.63	57	0.7
	Yes	2	50.00	0	0.00	2	50.00	4	
	Total	22	36.07	7	11.48	32	52.46	61	
Ear buzzing	No	22	37.93	7	12.07	29	50.00	58	0.24
	Yes	0	0.00	0	0.00	3	100.00	3	
	Total	22	36.07	7	11.48	32	52.46	61	
Sensorimotor changes	No	22	37.29	6	10.17	31	52.54	59	0.180
	Yes	0	0.00	1	50.00	1	50.00	2	
	Total	22	36.07	7	11.48	32	52.46	61	
Tremors	No	22	37.29	7	11.86	30	50.85	59	0.39
	Yes	0	0.00	0	0.00	2	100.00	2	
	Total	22	36.07	7	11.48	32	52.46	61	
Post-exertional pain	No	22	36.67	7	11.67	31	51.67	60	0.63
	Yes	0	0.00	0	0.00	1	100.00	1	
	Total	22	36.07	7	11.48	32	52.46	61	
Tachycardia	No	22	36.67	7	11.67	31	51.67	60	0.63
	Yes	0	0.00	0	0.00	1	100.00	1	
	Total	22	36.07	7	11.48	32	52.46	61	
Skin rashes	No	22	36.67	6	10.00	32	53.33	60	0.020
	Yes	0	0.00	1	100.00	0	0.00	1	
	Total	22	36.07	7	11.48	32	52.46	61	
Amnesia	No	22	36.67	7	11.67	31	51.67	60	0.63
	Yes	0	0.00	0	0.00	1	100.00	1	
	Total	22	36.07	7	11.48	32	52.46	61	
Sweating	No	22	37.29	7	11.86	30	50.85	59	0.39
	Yes	0	0.00	0	0.00	2	100.00	2	
	Total	22	36.07	7	11.48	32	52.46	61	
High blood pressure	No	22	36.67	7	11.67	31	51.67	60	0.63
	Yes	0	0.00	0	0.00	1	100.00	1	
	Total	22	36.07	7	11.48	32	52.46	61	
Constipation	No	22	36.67	7	11.67	31	51.67	60	0.63
	Yes	0	0.00	0	0.00	1	100.00	1	
	Total	22	36.07	7	11.48	32	52.46	61	
High temperature	No	22	36.67	7	11.67	31	51.67	60	0.63
	Yes	0	0.00	0	0.00	1	100.00	1	
	Total	22	36.07	7	11.48	32	52.46	61	
Blue skin	No	13	35.00	7	11.67	32	53.33	60	0.41
	Yes	0	100.00	0	0.00	0	0.00	1	
	Total	13	36.07	7	11.48	32	52.46	61	
Runny nose	No	13	35.59	7	11.86	31	52.54	59	0.84
	Yes	0	50.00	0	0.00	1	50.00	2	
	Total	13	36.07	7	11.48	32	52.46	61	
Anxiety	No	13	40.74	5	9.26	27	50.00	54	0.07
	Yes	0	0.00	2	28.57	5	71.43	7	
	Total	13	36.07	7	11.48	32	52.46	61	

Source: Questionnaire application results.

## Data Availability

The raw data supporting the conclusions of this article will be made available by the authors on request.
